# Nanoparticles: An Emerging Hope in Cancer Therapy

**DOI:** 10.3390/nano16090515

**Published:** 2026-04-24

**Authors:** Shahid Sher, Rosny Jean, Zaman Khan

**Affiliations:** 1 Department of Microbiology and Cell Science, University of Florida, Gainesville, FL 32611, USA; 2 School of the Environment, Florida Agricultural and Mechanical University, Tallahassee, FL 32307, USA; rosny.jean@famu.edu; 3 Department of Biological and Environmental Sciences, Faculty of Sciences, Emerson University Multan, Multan 60760, Pakistan; hmzamankhan@gmail.com

**Keywords:** nanoparticles, synthesis, cancer, drugs, delivery mechanisms, therapy

## Abstract

Cancer remains a major global health challenge, characterized by abnormal cell growth and metastasis. Current limitations of conventional therapies, particularly non-specific toxicity harming healthy cells, highlight the need for more targeted approaches. Nanotechnology offers a revolutionary solution, utilizing nanoparticles (NPs) for precise drug delivery to tumor sites while minimizing off-target effects. These nanometer-scale particles enable superior binding to cancer cell membranes, the tumor microenvironment, or nuclear receptors, facilitating significantly higher local concentrations of therapeutic agents. NPs, synthesized via physical, chemical, or biological methods, are categorized as organic (organic material-based) or inorganic (metallic particle-based). Key delivery mechanisms include the Enhanced Permeability and Retention (EPR) effect and Active Transport and Retention (ATR). This review specifically examines NP applications for the most prevalent cancers in the US (2025): breast, prostate, and lung. Gold and magnetic NPs show significant promise for early breast cancer detection. For lung cancer, polymeric NPs like PCL, PLA, and PLGA are effective carriers for peptides, proteins, and nucleic acids. BIND-014, a docetaxel-loaded NP formulation, represents an emerging strategy for prostate cancer. Clinically established examples include liposomal doxorubicin and albumin-bound paclitaxel. We comprehensively discuss the synthesis methods, delivery mechanisms, and the current landscape of NPs in research and clinical trials for these cancers. This analysis underscores the potential of nanotechnology to provide more effective and targeted therapeutic options for cancer patients in the future. A distinctive feature of this review is its comparative cancer-specific analysis of NP platforms in breast, prostate, and lung cancers. Unlike previous generalized reviews, this work integrates synthesis strategies, delivery mechanisms, translational challenges, and clinically relevant formulations to provide a bench-to-bedside perspective on the future of nanomedicine in oncology.

## 1. Introduction

Cancer is a major global health problem and represents one of the greatest challenges of the 21st century [1]. It is characterized by uncontrolled cell division driven by alterations in genetic, epigenetic, and transcriptional regulation [2]. Cancer development involves dynamic changes in the genome, leading to tumor formation. Tumors can be broadly classified as benign or malignant; benign tumors remain localized, whereas malignant tumors are invasive and can spread to distant organs via the bloodstream and lymphatic system, a process known as metastasis [3,4]. Cancer cells differ from normal cells due to deregulated signaling pathways, including those controlling the cell cycle, apoptosis, DNA repair, and redox balance [5]. The development of cancer typically progresses through three stages: initiation, promotion, and progression. During initiation, DNA damage caused by carcinogens (e.g., radiation, chemicals, and viruses) leads to activation of oncogenes and inactivation of tumor suppressor genes [6]. Subsequent stages involve uncontrolled proliferation and eventual metastasis. Importantly, cancer arises from the accumulation of multiple genetic alterations, as described in the multi-hit hypothesis proposed by Nordling and Knudson [7,8].

Cancer is currently one of the leading causes of premature death worldwide [9]. Despite significant advances in diagnosis and treatment, the global burden of cancer continues to rise [10]. In 2012, approximately 14 million new cancer cases and 8.2 million deaths were reported globally, and this number is expected to increase substantially in the coming decades [11]. It is projected that cancer incidence could reach 24 million new cases within the next 20 years, with nearly 40% of individuals being diagnosed with cancer during their lifetime. Furthermore, cancer-related mortality is estimated to rise dramatically, reaching approximately 30 million deaths annually by 2030, representing a nearly 200% increase compared to earlier decades [12,13,14]. These alarming statistics highlight the urgent need for more effective and targeted therapeutic strategies.

One of the major limitations in current cancer therapy is the development of drug resistance in solid tumors. This resistance arises from multiple mechanisms, including alterations in gene expression, activation of drug efflux pumps, deregulation of DNA repair pathways, and impaired apoptotic signaling. In addition to these cellular mechanisms, the tumor microenvironment (TME) plays a critical role in determining therapeutic outcomes by influencing drug penetration, cellular uptake, and retention within tumor tissues. The TME is highly heterogeneous and is characterized by abnormal vasculature, hypoxia, acidic pH, and a dense extracellular matrix, all of which act as physical and biological barriers to effective drug delivery. Recent studies have further emphasized that tumor heterogeneity and microenvironmental complexity significantly limit the efficacy of conventional therapies and nanoparticle-based systems [15,16]. Therefore, overcoming these barriers remains a major challenge in improving cancer treatment outcomes.

Recent advances in nanomedicine have demonstrated that prodrug-based nanoassemblies can significantly enhance the therapeutic efficacy and safety of conventional chemotherapeutics. For instance, reduction-responsive epirubicin (EPI) nanoassemblies have shown improved pharmacokinetics, controlled drug release, and reduced systemic toxicity, with β-ESC nanoassemblies exhibiting superior antitumor activity. These findings highlight the potential of rational nanoparticle design in overcoming key limitations of traditional chemotherapy [17]. Recent studies have further demonstrated that biomimetic and carrier-free nanoparticle systems can enhance therapeutic targeting and safety. For example, macrophage membrane-coated nanoassemblies combining natural compounds and conventional drugs have shown improved site-specific accumulation, anti-inflammatory effects, and reduced toxicity in disease models. Such strategies highlight the potential of biomimetic nanocarriers to overcome limitations of traditional drug delivery systems [18].

Although numerous recent review articles have summarized nanoparticle synthesis strategies and their general applications in oncology, most remain largely platform-oriented, focusing on material classes such as liposomes, polymeric nanoparticles, dendrimers, and metallic systems. However, these reviews often lack a critical evaluation of how nanoparticle physicochemical properties interact with tumor-specific heterogeneity, biological barriers, and microenvironmental conditions across different cancer types. In particular, limited attention has been given to comparative, cancer-specific analyses that address variations in vascular architecture, stromal density, immune interactions, and drug resistance mechanisms among major solid tumors. Furthermore, translational challenges, including clinical efficacy, delivery limitations, and variability in patient response, are frequently discussed in isolation rather than in an integrated framework. Therefore, this review addresses these gaps by providing a comparative and cancer-specific perspective focused on breast, prostate, and lung cancers, integrating nanoparticle design, tumor biology, delivery barriers, and clinical translation [16].

This study was conducted as a narrative review aimed at critically summarizing the current landscape of nanoparticle-based cancer therapy. The relevant literature was identified through structured searches of PubMed, Scopus, Web of Science, and Google Scholar using keywords such as nanoparticles, cancer therapy, nanomedicine, targeted drug delivery, tumor microenvironment, EPR effect, clinical translation, breast cancer, prostate cancer, and lung cancer. Articles were selected based on thematic relevance, scientific quality, translational significance, and recency, with priority given to recent review articles, preclinical studies, clinical trials, and clinically approved nanomedicine formulations. Although this review does not follow a formal systematic review protocol (e.g., PRISMA guidelines), efforts were made to ensure comprehensive coverage and balanced representation of the current literature. Additionally, landmark studies were included where necessary to provide historical context for key concepts such as the enhanced permeability and retention (EPR) effect.

## 2. Limitations in Conventional Cancer Therapies

There are several causes of cancer, including radiation exposure, environmental pollutants, and unhealthy lifestyle factors such as poor diet, lack of physical activity, smoking, and stress; however, only a small proportion (5–10%) is attributed to inherited genetic factors [19,20]. Traditionally, cancer treatment relied on surgery and radiotherapy, but modern approaches now include chemotherapy, proton therapy, laser therapy, photodynamic therapy, cryotherapy, differential therapy, and sentinel lymph node biopsy [21]. Despite these advancements, many conventional therapies remain non-specific and affect both cancerous and healthy tissues. For example, chemotherapy lacks selectivity and often damages normal cells, leading to adverse effects such as neurotoxicity and increased risk of venous thromboembolism [22,23]. Similarly, proton therapy and thermotherapy may also impact healthy tissues, while gene therapy faces challenges in delivering therapeutic genes to all tumor cells effectively [24,25,26,27]. These limitations highlight the need for more targeted and less toxic treatment strategies.

In addition to systemic toxicity, conventional cancer therapies are significantly limited by multidrug resistance, poor tumor selectivity, and insufficient penetration into heterogeneous tumor tissues. These issues arise from alterations in gene expression, activation of drug efflux mechanisms, deregulation of DNA repair pathways, and impaired apoptotic signaling [28]. As a result, therapeutic efficacy is often compromised, leading to reduced treatment success and poor patient outcomes. These persistent challenges have accelerated the development of advanced delivery systems aimed at improving tumor-specific targeting and minimizing off-target effects [29].

Nanotechnology has emerged as a multidisciplinary field with significant potential to revolutionize cancer therapy. It involves the design and application of nanoparticles with dimensions typically below 100 nm, which exhibit unique physicochemical properties compared to their bulk counterparts [30]. In oncology, nanotechnology enables improved diagnosis, detection, and treatment strategies, collectively referred to as nano-oncology [31]. Nanoparticles offer several advantages over conventional therapies, including enhanced drug solubility, improved bioavailability, controlled drug release, multifunctionality, and reduced systemic toxicity [28].

One of the key advantages of nanoparticles is their ability to accumulate preferentially in tumor tissues through the Enhanced Permeability and Retention (EPR) effect, which arises from leaky tumor vasculature and impaired lymphatic drainage [32,33]. However, the EPR effect is highly variable across tumor types and even within different regions of the same tumor. Differences in vascular density, stromal composition, extracellular matrix stiffness, and interstitial fluid pressure significantly influence nanoparticle distribution and penetration, resulting in heterogeneous and often unpredictable delivery outcomes in clinical settings.

Nanoparticles coated with polyethylene glycol (PEG) can reduce opsonization and help evade immune system recognition [34]. However, upon exposure to biological fluids, nanoparticles rapidly adsorb proteins on their surface, forming a protein corona that alters their physicochemical and biological identity [35,36]. This acquired corona can mask targeting ligands, modify surface charge, and increase hydrodynamic size, thereby reducing receptor-mediated cellular uptake. In addition, opsonin-rich coronas promote recognition by macrophages and the mononuclear phagocyte system, leading to accelerated immune clearance, shortened circulation time, and reduced tumor accumulation.

To reach tumor sites, nanoparticles must overcome multiple biological and physical barriers, including epithelial and endothelial layers, cellular membranes, enzymatic degradation, and interactions within systemic circulation. These interactions can significantly alter nanoparticle behavior, reducing targeting efficiency and therapeutic precision [33]. Such barriers limit effective drug delivery and highlight the complexity of nanoparticle transport within the body.

For optimal therapeutic performance, nanoparticles must be carefully engineered with appropriate size, surface chemistry, and biocompatibility. They should remain stable in circulation, evade clearance by the reticuloendothelial and mononuclear phagocyte systems, accumulate efficiently within the tumor microenvironment, and penetrate tumor tissues effectively. Furthermore, successful delivery requires nanoparticles to reach specific intracellular targets while maintaining controlled drug release, necessitating precise optimization of their physicochemical properties.

Despite their significant advantages, nanoparticle-based systems still face major limitations that hinder their clinical translation. A key challenge is the variability of the EPR effect, which leads to inconsistent nanoparticle accumulation and therapeutic outcomes. Highly fibrotic or poorly vascularized tumors often exhibit weak nanoparticle penetration, resulting in reduced efficacy. These limitations underscore the need for tumor-specific nanoparticle design and integrated strategies to overcome biological barriers and improve delivery efficiency in heterogeneous cancers [37].

## 3. Synthesis of Nanoparticles

**Figure 1:**
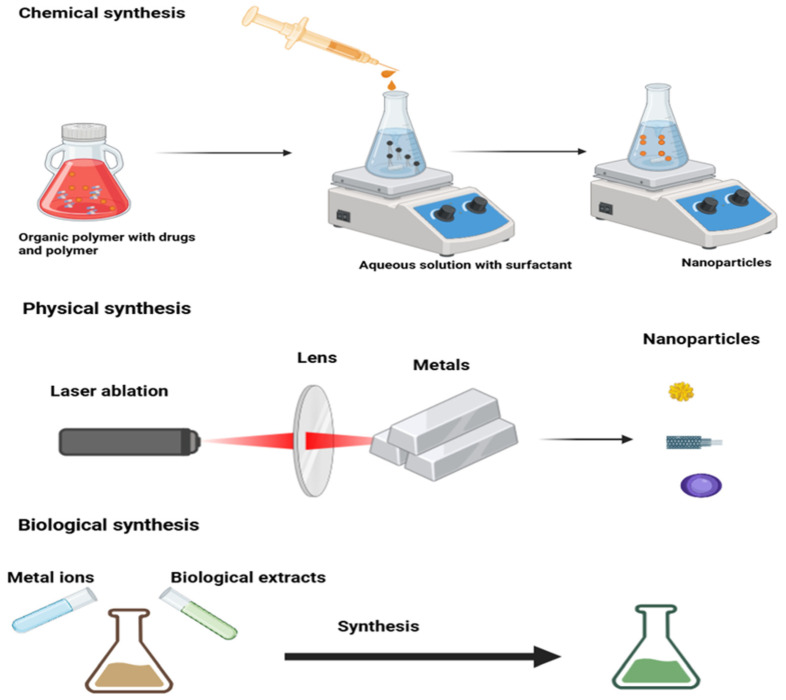
Synthesis of nanoparticles via three methods: chemical, physical, and biological (Created in Biorender).

There are two approaches for the synthesis of nanoparticles: the first one is a bottom-up approach, and the second one is a top-down approach [38]. The first one is a constructive method in which nanoparticles are built from atoms to clusters and then NP, while in the second approach, which is a destructive method in which we convert bulk materials to NPs; it comes under physical preparation of NP synthesis. There are two further ways to synthesize nanoparticles: chemical and biological [39]. All three synthesis methods are shown in Figure 1. Both bottom-up and top-down approaches come under chemical methods. The first way of synthesizing has some advantages due to the absorption of some toxic chemicals on the surface of nanoparticles. In the biological way of synthesis, we used microorganisms [40,41], enzymes [42], fungi [43], plants, and the extract from plants [44,45]. The biological way of synthesis is eco-friendly, which is unlikely to be a chemical method. This biological way is still evolving and has an application in various sectors, from antibacterial to drug delivery and in cancer treatment, too, such as silver nanoparticles.

Both prokaryotes and eukaryotes are being used for the synthesis of nanoparticles such as silver, gold, zirconium, palladium, platinum, iron, and oxides of metals such as zinc oxide and titanium oxide. Microorganisms such as bacteria, fungi, algae, and actinomycetes produce intracellularly or extracellularly, meaning on the location of the nanoparticles inside or outside of the organisms. In intracellular ways of synthesis, metal ions go inside the organisms and form nanoparticles, which are usually smaller in size [46]. In an extracellular way of synthesis, organisms secrete these nanoparticles into their extracellular surroundings have more applications compared to the first one, and these nanoparticles are usually devoid of other intracellular compartments of cells [46]. Both bacteria and fungi have a role in the biological generation of nanoparticles. The latter one is of much importance due to tolerance and bioaccumulation of metallic ions [47]. For the synthesis of polymer-based nanoparticles (PGMD-CUR NPs), the nanoprecipitation method was used [48]. This method is also called the solvent displacement method, in which precipitation of a polymer is carried out from an organic to an aqueous medium, which takes place in the presence of surfactants. The synthesis of NPs through nanoprecipitation is less laborious and economical. After the synthesis of NPs through nanoprecipitation, which usually requires two-step centrifugation, 2400× *g* and supernatant, followed by 16,000× *g* for 30 min, the pellet should be washed and resuspended in dd water. NPs need to be analyzed through FTIR spectroscopy for the evaluation of the interaction between the polymer and drug.

All these synthesis methods have various advantages and disadvantages in oncology applications. The precise size and morphology of NPs can be achieved through physical methods, and this has a positive impact on reproducibility in drug delivery and targeting tumors. The highly expensive instrumentation and energy input make the physical method restricted. Excellent scalability and surface functionalization flexibility can be achieved through chemical synthesis, and this makes it highly suitable for ligand-mediated cancer cell targeting and bioavailability enhancement; however, concerns related to toxic solvent residues and biocompatibility remain. In contrast to chemical methods, the biological synthesis methods are eco-friendly and produce highly biocompatible nanoparticles with less toxicity, which is predominantly useful for translational oncology, while batch-to-batch changeability and restricted control over particle homogeneity may limit certain applications [38,49].

## 4. Nanoparticles: A Vehicle for Cancer Drugs

NPs are nanocarriers for cancer drugs or medicine. The pharmacokinetic values of drugs have been improved in terms of high efficacy and fewer side effects due to NPs [50]. For drug delivery to cancer cells, the structure of NPs can be made with various materials such as metallic particles, lipids, polymers, and these can be made in various sizes and shapes [51]. The application of drug delivery systems is increasing day-by-day. In the future, there is a need to focus more on multiple functions of NPs, such as the delivery of drugs and simultaneous imaging [52,53]. DDS are the repository for drugs that are very specific in releasing the drugs at the proper time and locations [54]. The structures of NPs have some unique characteristics that control the drug release process in the body and some other phenomena. In the last half-century, numerous improvements have been carried out in the field of polymer, physics, biology, chemistry, and mechanics, which have affected the variability of nanocarriers, and various sorts of carriers have been introduced in the field of medical sciences [55].

## 5. Classifications of NPs

Nanoparticles used in oncology can be systematically classified in a hierarchical manner based on (i) material composition, (ii) functional properties, and (iii) therapeutic modality. At the material level, nanoparticle systems are broadly divided into organic nanoparticles (liposomes, solid lipid nanoparticles, polymeric nanoparticles, micelles, dendrimers, polymersomes, and hydrogels), inorganic nanoparticles (metallic nanoparticles, silica nanoparticles, magnetic nanoparticles, carbon nanotubes, quantum dots, and ceramic systems), and hybrid nanoparticles that integrate multiple material platforms. At the second level, these systems can be distinguished by functional features such as biodegradability, tumor targeting, stimulus responsiveness, imaging capability, and stealth surface modification. Finally, their therapeutic roles include applications in chemotherapy, nucleic acid delivery, photothermal therapy, imaging, immunotherapy, and multimodal theranostics [56].

**Figure 2:**
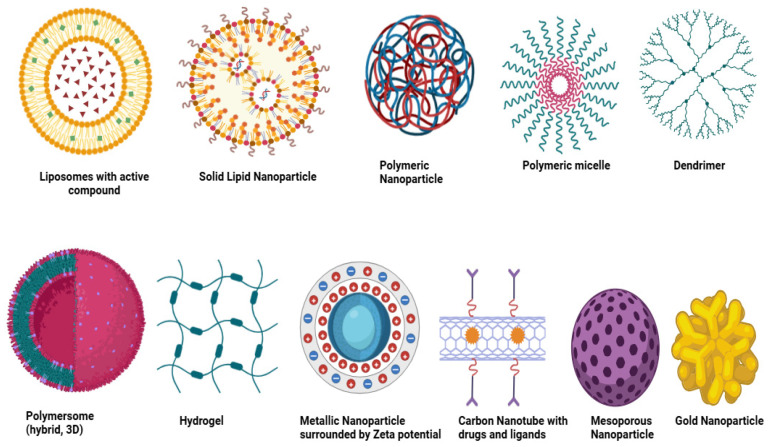
Different types of Nanoparticles (created in Biorender).

Nanoparticles used as drug carriers can be broadly categorized into organic and inorganic systems. Organic nanoparticles are composed of biodegradable and biocompatible materials, whereas inorganic nanoparticles are primarily based on metallic or mineral components with unique physicochemical properties. Different types of nanoparticles are illustrated in Figure 2.

### 5.1. Organic Nanoparticles

Liposomes are among the earliest nanoparticle systems, first described by Bangham and Horne in 1964 using electron microscopy [57]. These vesicular structures are composed of amphiphilic phospholipid bilayers with an aqueous core, closely resembling biological membranes [58]. Their size, composition, and lamellarity can be easily modulated, allowing efficient encapsulation of both hydrophilic and hydrophobic molecules [59]. Liposomes have been widely applied in drug delivery and vaccine development [60,61,62,63], with liposomal doxorubicin (Doxil^®^) being approved for ovarian cancer and Kaposi sarcoma in 1995 [64].

Solid lipid nanoparticles (SLNs) were first introduced by Muller and Lucks in the 1990s [64]. These nanoparticles remain solid at both room and physiological temperatures and are composed of lipids such as triglycerides, fatty acids, steroids, and waxes [65]. Typically smaller than 1 μm, SLNs offer improved stability and controlled drug release, with administration possible via oral and injectable routes [50,66].

Polymeric nanoparticles are widely used due to their stability, scalability, and tunable properties. These systems are composed of biodegradable and biocompatible polymers and can exist in vesicular or matrix structures, enabling diverse drug-loading strategies [67,68]. Abraxane^®^, a paclitaxel-based nanoparticle formulation, is a clinically approved example that avoids toxic solvents associated with conventional formulations [69]. Their biological performance is strongly influenced by size, surface charge, composition, and degradation behavior. Nanoparticles in the range of 50–200 nm generally show improved circulation and tumor accumulation, whereas smaller particles enhance penetration and cellular uptake. Surface charge affects nanoparticle–cell interactions, while polymer composition and degradation kinetics regulate drug release and biocompatibility [70,71].

Polymeric micelles are formed through the self-assembly of amphiphilic block copolymers into core–shell structures capable of solubilizing hydrophobic drugs [60]. Their surfaces can be functionalized for targeted delivery, and clinically relevant formulations such as NK105 and NK911 contain paclitaxel and doxorubicin, respectively [61,62].

Dendrimers are highly branched, tree-like macromolecules with well-defined size and structure [63]. Their surfaces can be chemically modified to enable drug conjugation and targeting. Early synthesis was reported by Vögtle and Tomalia in the 1970s [64], and Vivagel^®^ is a notable example used as an antiviral agent against HIV and herpes viruses [65].

Polymersomes gained more attention as a versatile carrier in the last few decades due to their colloidal stability and ability to integrate various kinds of molecules and drugs. The retention times of Ps can be increased by conjugating polyethylene glycol with a block copolymer. Numerous Ps have been developed in medical imaging and drug delivery, apart from the nanoreactors and electronics sectors. These are artificial vesicles made of synthetic amphiphilic block copolymers [66,67,68]. It contains hydrophilic molecules in the aqueous core and hydrophobic drugs in bilayer membranes. It can load multiple drugs; its robustness of membrane stealth properties is a core part of research for the application of drug delivery [69,72].

Hydrogel NPs have 3D structures; they are used for encapsulating and delivering drugs. These structures have a biosorbent sort of nature, absorb water, and swell in an aqueous environment [73]. They release the drugs due to an alteration in pH and temperature [74]. This kind of system is being used for the delivery of proteins and DNA [75].

### 5.2. Inorganic Nanoparticles

Mineral NPs: they have a nucleus in the center with fluorescent, magnetic, and electric properties, and are coated by organic materials and give protection to the inner part from external degradation. This outer layer can further bind with molecules having positively charged, amine or thiol chains via covalent or electrostatic bonds [76].

Quantum dots are fluorescent with a 2–10 nm size, having hundreds to thousands of atoms, including cadmium, zinc, selenium, technetium, tantalum, and indium [77]. Based on carbon, it is further divided into three different categories: graphene quantum dots, carbon, and non-diamond quantum dots. Due to rapid excretion and biocompatibility, the graphene quantum dots are commonly used. The optimization of quantum dots is the biggest challenge. For prostate cancer aptamer–doxorubicin is being used [78]. Carbon NPs are being used in medical arenas at a large scale due to their electronic, optical, and mechanical properties, along with biocompatibility [79,80].

Carbon-based NPs are further categorized into graphene (a 2D crystal with sp2 hybridization of carbon), carbon nanotubes (single or multi-walled walls cylindrical in shape, discovered in 1980), carbon nano horns, fullerenes (large carbon-cage molecules with various conformational spheres, ellipsoids, or tubes), and graphene [38].

Metallic nanoparticles: they have common use in DDS and biological imaging due to their unique magnetic, optical, and photothermal properties. Silver, gold, copper, and iron-based NPs are commonly used [38]. Gold NPs are being used to target the intracellular drug carrier due to their surface properties and size [81]. For the detection of nodal metastases, Combidex^®^ (iron-based NPs) is in its final stage of clinical trials. Feraheme^®^, another iron-based NP, was used for the treatment of anemia, nodal metastases in prostate cancer, and testicular cancer as per the FDA [82,83].

Silica NPs are good ones due to the presence of silicon in natural materials. Mesoporous silica is considered one of the ideal carriers for drugs due to its pharmacokinetic properties. The previous study indicated the successful uptake of mesoporous silica containing camptothecin by colorectal cancer cells [38].

Magnetic NPs, commonly used for MRI imaging and DDS for metals and their oxides. For breast cancer, the most effective magnetic NPs are LHRH-conjugated superparamagnetic iron oxide NPs [84]. Feridex^®^ and Resovist^®^ are commercially available magnetic NPs for the treatment of metastases of liver and colon cancer [85].

Beyond material composition, the therapeutic behavior of nanoparticles is strongly influenced by functional characteristics such as size, surface charge, targeting ligands, biodegradability, and stimulus-responsive release mechanisms. These properties determine circulation half-life, tumor penetration, cellular uptake, and compatibility with specific treatment modalities [72].

From a therapeutic perspective, nanoparticle platforms can also be categorized according to their principal oncologic application, including drug delivery, gene therapy, imaging, photothermal therapy, radiosensitization, and combination treatment strategies. This modality-based perspective helps align nanoparticle design with specific clinical objectives [80].

## 6. Delivery Mechanisms for NPs

**Figure 3:**
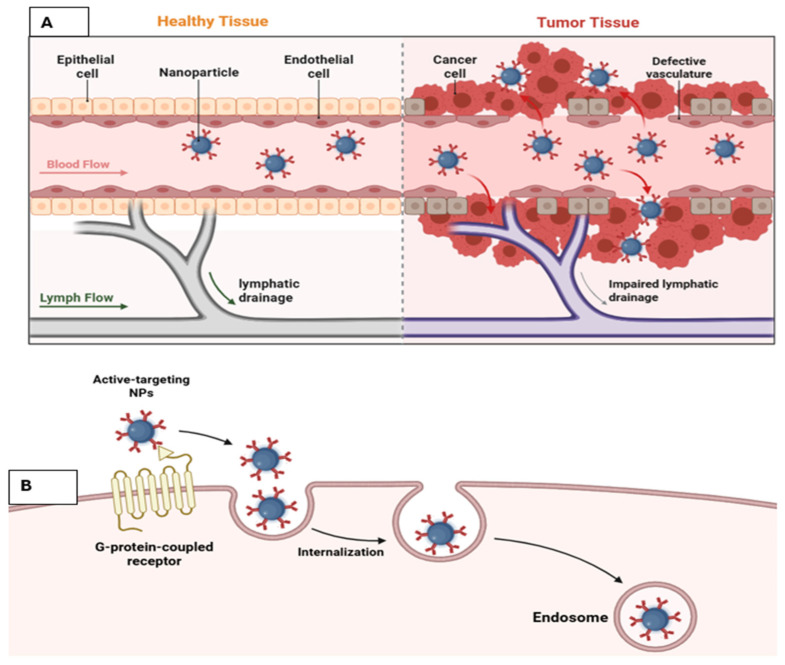
Proposed illustration of nanoparticle delivery mechanism: (**A**): Passive targeting of nanoparticles to the cancer cells, (**B**): active targeting of nanoparticles to cancer cells (created in Biorender).

The mechanism involved in nanoparticle delivery to solid tumors is not well understood. In general, there are two mechanisms for the delivery of NPs into cancer cells (Figure 3). One is passive and the other is active. The first one is also called the enhanced permeability and retention (EPR) effect, which states that a gap between endothelial cells is the route for NPs entrance, which enters passively, and NPs retain themselves due to poor lymphatic drainage [86,87,88]. There is no exit route for NPs in passive mechanisms; as a result, they remain inside the tumor. For EPR, the important factors are the size and time for circulation by NPs in the blood system [89]. The size of the NPs should be smaller than the gap size in interendothelial gaps. Alternatively, another mechanism suggested that NPs enter through an active transport system passing by endothelial gaps and are retained in cells due to interactions with cancer cells’ components, and lymphatic vessels are the way for their exit.

Globally, only 30 nanomedicines are available, which are approved clinically, and mostly nanomedicine almost 86% of nanomedicines fail in the clinical trials phase 3 due to less therapeutic efficacy [90]. The failure attempts in nanomedicine trigger a debate among scientists on the association of the EFR effect in NP delivery to the tumor [15,91,92,93]. It has been reported that macromolecules and nanoparticles can accumulate and remain in the tumor. In 1900, scientists agreed that cancer cells accumulate more colloidal dyes than healthy cells [94]. Later, cancer cells were subjected to accumulated proteins, radioactive labels, crystalline substances, colloidal carbon, and iron oxide colloids [95,96,97,98]. These studies gave a way to inject particulates into the tumor and accumulate there. In 1984, for the first time SMANCS–lipiodol was accumulated and retained in the tumor and explained via the EPR effect [99]. SMANCS is an anticancer drug conjugated from neocarzinostatin (NCS) with a synthetic polymer (styrene-co-maleic acid) and is soluble in lipophilic solvents. The experimental evidence for tumor blood vessel permeability was analyzed in the LS174T tumor in mice, in which 90 nm liposomes were accumulated in mice tumor; this was due to an inter-endothelial gap in blood vessels [100]. The biggest disadvantage of the EPR effect is the exit pathway for NPs from solid tumors, which is lacking, impaired, and defective [101,102,103,104]. The lumen of lymphatic vessels in tumor cells becomes collapsed and small, which does not allow the NPs to go outside [86,87]. Despite its foundational role in nanomedicine, the EPR effect remains highly variable and often clinically inconsistent. Its efficiency is strongly influenced by tumor vascular heterogeneity, extracellular matrix density, stromal fibrosis, elevated interstitial fluid pressure, and differences in lymphatic collapse among tumor types. These factors can substantially reduce nanoparticle penetration beyond perivascular regions, resulting in non-uniform intratumoral distribution and poor reproducibility of therapeutic outcomes across patients. Therefore, reliance on passive accumulation alone is increasingly recognized as a major translational limitation in solid tumor nanotherapy [86].

The other mechanism for NP delivery in the tumor is active transport and retention (ATR). Which was proposed very recently in 2023 [87]. ATR stated that NPs enter tumor cells through active transport involving endothelial cells, are retained in the tumor due to interaction with cellular/cellular compartments, and exit through the lymphatic system. This process involved energy to transfer NPs from blood vessels to tumors. Furthermore, there is involvement of transcytosis pathways (phagocytosis, macropinocytosis, caveolar-mediated and clathrin-mediated endocytosis, and caveolar-independent and clathrin-independent mechanisms) [105]. ATR phenomena were reported in gold NPs with different sizes (15, 50, and 100 nm) [106]. Transcytosis, as well as Vesiculo-vacuolar organelles, can also transport gold NPs conjugated with albumin and liposome-silica 60 nm into the tumor cell [107,108]. For the exit of NPs from tumor cells, APR suggested that it happens through intratumorally and peritumoral spaces. In one of the studies, it has been shown that silica NPs (100 nm), Liposomes NPs (100 nm), and gold NPs of different sizes can exit from tumors B16F10, 4T1, and MMTV-PyVT, and 45% of the total gold NPs of 15 nm can exit after 5 days [109]. Despite their importance in nanoparticle-based cancer delivery, both the enhanced permeability and retention (EPR) effect and active transport and retention (ATR) mechanisms face significant limitations in heterogeneous tumors. Variations in vascular permeability, stromal density, interstitial fluid pressure, and endothelial transcytosis across different tumor regions and among patients can markedly reduce nanoparticle penetration, retention, and therapeutic consistency. These challenges are particularly pronounced in poorly vascularized, metastatic, or fibrotic tumors, where reliance on EPR or ATR alone may result in suboptimal drug delivery outcomes.

Although the ATR mechanism provides a more dynamic explanation for nanoparticle transport, several mechanistic uncertainties remain unresolved. The contribution of specific transcytosis pathways varies significantly according to nanoparticle size, surface chemistry, protein corona composition, endothelial phenotype, and tumor metabolic status [110]. In addition, active transport processes are energy-dependent and may be impaired in hypoxic or poorly perfused tumors, limiting delivery efficiency. These mechanistic complexities highlight the need for standardized in vivo models capable of distinguishing true endothelial transcytosis from passive extravasation and non-specific cellular uptake [111]. Taken together, these findings indicate that successful nanoparticle delivery depends not only on passive or active transport mechanisms but also on overcoming multiple biological barriers, including protein corona formation, immune clearance, stromal density, and heterogeneous tumor perfusion. Future nanoparticle systems must therefore be designed with mechanism-aware optimization strategies that integrate transport biology, physicochemical tuning, and tumor-specific microenvironmental constraints [110,111].

A comparative evaluation of nanoparticle platforms shows that their clinical potential depends on balancing efficacy with safety, stability, and translational feasibility [16]. Lipid-based nanoparticles and liposomes have achieved the greatest clinical success because they generally offer favorable biocompatibility, reproducible manufacturing, and reduced systemic toxicity, whereas polymeric nanoparticles provide excellent tunability and controlled release but may vary in degradation behavior and formulation complexity. Inorganic nanoparticles, including gold, magnetic, and silica-based systems, demonstrate strong imaging and theranostic value, but concerns regarding long-term retention, biodegradability, and potential toxicity still limit broader clinical adoption. Therefore, the most promising nanoparticle systems are not necessarily those with the highest experimental anticancer activity alone, but those that also demonstrate acceptable safety, formulation stability, scalable production, and clear clinical relevance [16,72].

Some major kinds of cancer that are high in terms of prevalence and incidence around the globe are breast cancer in women, lung and stomach cancer in both males and females, and esophagus and prostate cancer in men [112]. The mortality in the lungs, liver, stomach, and pancreas is greater than in other sorts of cancer, while colorectal cancer is equally lethal for both populations and is the second leading cause of cancer [112].

## 7. Nanoparticles in Breast Cancer

**Figure 4:**
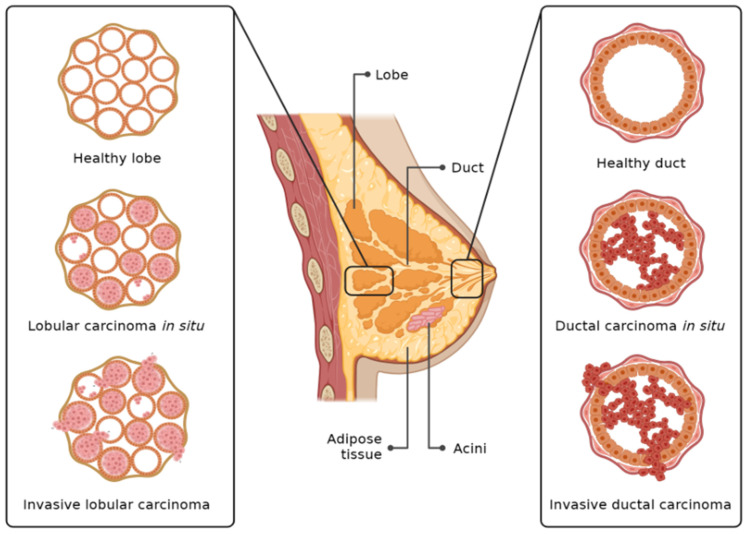
Graphical representation of breast cancer.

Carcinoma of the breast is among the list of common cancers in females, which accounts for almost 15% of all cancer mortalities and 23% of all cancer incidence [113]. The incidence and mortality associated with breast cancer have been reduced after proper screening and the advancement of treatment [114]. In the pathophysiology of breast cancer, there is involvement of different factors such as environmental, genetic, and lifestyle. The genes BRCA1 and BRCA2 can contribute almost 10% to mammary gland carcinoma [115]. Globally, 20% of breast cancer is due to an unhealthy lifestyle [116]. The breast cancer graphical illustration is shown in Figure 4. However, we still have a lot of challenges ahead for breast cancer patients; one is the adverse effects associated with treatment. To overcome these challenges, scientists have introduced nanotechnology for the early diagnosis and treatment of breast cancer [117].

Conventional therapies in breast cancer, such as surgery, radiotherapy, and chemotherapy, can reduce relapse and manage this cancer with some significant side effects to healthy cells, which include multiple drug resistance, low specificity, low retention time, and toxicity of the healthy cells [118]. Nanotechnology is being used against breast cancer in a variety of methods, including targeted delivery of drugs, targeted radiation therapy, and cancer monitoring with emerging imaging technologies. In short, nanotechnology has huge potential in cancer detection and treatment. Imaging of breast cancer can be carried out with MRI [119]. Gold and Magnetic NPs are the best option for the early detection of breast cancer, as these NPs are getting more attention from scientific communities [113,120]. These both act as contrasting agents for MRI due to their nano-size and higher magnetism [121]. The range of MNP diameter is 1–1000 nm [122]. Cobalt, nickel, iron, and their oxides, such as hematite, cobalt ferrite, ferric oxide, form different magnetic elements in MNPs [123]. Fe_3_O_4_ MNPs loaded with tamoxifen (used to treat hormone receptor-positive breast cancer and has some side effects if used independently) and coated with tyrosine were prepared against breast cancer [124]. The biocompatibility of this formulation of MNPs was checked against RBCs, and in vitro toxicity was determined against the MCF-7 cell line, but animal trials are underway for these MNPs. Artemisinin (an antimalarial agent) is also effective against cancer. MNPs are embedded with artemisinin and coated with chitosan, which increases their entrapment efficacy up to 62.5% due to the coating of chitosan. These MNPs enhanced the drug targeting in mice with 4T1 breast cancer [122]. MNPs including peel extract of black pomegranate (BPPE), coated with chitosan (containing a functional group for attachment with drugs), have significantly increased loading efficiency to 61.0%. These MNPs loaded with drugs were effective against 4T1 cancer cells compared to free drugs [125]. Carnosine-coated MNPs are more effective against breast cancer [126]. Disulfiram-loaded MNPs against MCF-7 breast cancer has greater cytotoxic effects [127]. MNPs form an aggregate due to high surface tension, which is a drawback for MNPs, but coating with surfactants and polymers can reduce their clumping [128]. Disulfiram, an anticancer drug for breast cancer, has been extensively used, but its insolubility in water and early breakdown make it less effective for breast cancer treatment. But loading this drug into mesoporous silica-coated MNPs having folic-acid-conjugated poly-ethyleneimine made it more effective against MCF-7 breast cancer, evaluated by MTS assay. The coating of mesoporous silica enhances surface area for biocompatibility and water dispersibility [127]. The oxide of iron MNPs are good drug carriers and highly cytotoxic to breast cancer cells [129]. In one of the studies, it was reported that fluorouracil-imprinted polymer-coated Fe_3_O_4_ MNPs are effective against breast cancer, in an experiment conducted on female Balb/C mice having breast cancer [130]. Idarubicin, an anticancer drug, ruptures cancer cells by inhibiting the DNA and membrane of the cell [131]. But at the same time, it has some adverse effects, such as neutropenia; bone marrow suppression, which can be managed by PEG-coated idarubicin-loaded MNPs conjugated with folic acid, which increase its retention time; solubility in water; and biocompatibility [132]. In another study, Fe_3_O_4_ MNPs were coated with citric acid, having a functional group for further conjugation with Polyamidoamine (PAMAM) dendrimers and curcumin (having anticancer properties), further encapsulated in a PAMAM layer is effective in breast cancer [133].

In addition to chemotherapy, another approach is hypothermia, which eliminates cancer cells via heating by irradiation, magnetic hyperthermia, ohmic heating, and microwave [134]. Hatamie et al. developed cobalt ferrite MNPs, which were further enclosed in graphene oxide against breast cancer, which has strong hyperthermic activity [135]. Trastuzumab, an anticancer drug further coated with PEG-MNPs, showed a very specific hypothermic activity against HER2^+^ cancer cells [136]. An experiment was conducted to compare the efficacy of hypothermia induced by microwave and magnetic nanocarriers (ferromagnetic hydroxyethyl starch-coated MNPs) against MTGB breast tumors showed a very similar sort of efficacy, followed by histopathology of the mouse model, but the magnetic MNPs did less damage to healthy surrounding cells due to the localized heating effect [137]. Breast cancer NPs and clinical trials against different models, along with their mechanisms, as shown in Table 1.

## 8. Nanoparticles in Lung Cancer

Lung cancer accounts for almost 23% of mortality associated with cancer, which is more common than colon, prostate, and breast cancer [113]. The high fatality rate in lung cancer is due to a lack of approaches in early diagnosis. Because of histological appearance, there are two classifications of lung cancer, one is small-cell lung cancer (SCLC) and the second one is non-small-cell lung cancer (NSCLC); the first one is more lethal than the second if not treated very well. The mutagenic substance in cigarette smoke is the causative agent for both types of lung cancer [148]. Apart from smoking, the genetic alterations in genes and infection from H. pylori could also trigger lung cancer [149]. Currently, however, the approaches we have, such as chemotherapy and radiotherapy, only work in the early stage of lung cancer [150]. For the complete eradication of lung cancer, there is a need to implement nanoscale materials to get a better cure for cancer. Several studies have shown that intratracheal delivery of nanogel or nano spray confirmed effective outcomes compared to the parenteral route. The delivery of anticancer cells to lung cells was tested by chitosan-based nanogels delivered to the pulmonary system and are an effective way of delivering them [151]. The most commonly used polymers for lung cancer are poly(ϵ-caprolactone) (PCL), polylactic acid (PLA), poly(lactide-*co*-glycolide) (PLGA) (a biodegradable polymer approved by the FDA, which can be used for delivery of peptides, proteins, and nucleic acids), alginic acid, gelatin, and chitosan [152]. In an experiment, a mouse was injected with CT26 cells (a type of colon cancer cell line) first, then the pCMV-Muβ-encoding plasmid was loaded on chitosan, which was in dry powder form, and exhibited an interesting outcome [153]. A study was conducted regarding camptothecin, a water-insoluble anticancer drug. 10-hydroxycamptothecin and 7-butyl-10-aminocamptothecin were loaded on a polyester dendrimer (biocompatible) and validated against four different cancer cells (breast adenocarcinoma, non-small cell lung carcinoma, colorectal adenocarcinoma, and glioblastoma cells). These anticancer compounds exhibited good activity in terms of uptake by cancer cells as well as their retention in the tumor [154]. A study conducted by Guthi et al. exhibited that LCTP can specifically bind to α_v_β_6_-integrin (a surface protein in the H2009 cell line). He designed a multifunctional PEG-*b*-PDLLA (poly(d,l-lactide) micelle system with LCTP and loaded SPIONs, Super-paramagnetic iron-oxide nanoparticles with Doxo. SPIONs are tiny bits of iron that show up clearly in an MRI scanner [155]. In 1999, a group of scientists closely working with Guilford Pharma designed a paclimer, small plastic beads also called polylactofate, with 10% of its size containing paclitaxel (PTX), and these beads release PTX in a time manner and were effectively tried against NSCLC [156]. A549 lung cancer cells were destroyed with a photothermal therapeutic agent, which was developed with hollow Au/Ag nanostructures with a dendritic morphology [157]. In addition to this, LL2 (Lewis lung carcinoma) cytotoxicity was generated after treatment with Au NPs in conjugation with an analog of folic acid, which is methotrexate [158]. Imaging agents (organic dye (FITC), and a tumor-targeting antibody (Ab CD-10)), along with silica and multifunctional cobalt ferrite, a magnetic nanoparticle. The outcome showed that A549 lung cancer cells took it specifically [159]. The rare earth element named neodymium has cytotoxic activity against cancer cells [160]. The biomedical application of nanodiamond (ND), including the tracking and labeling of cancer cells, has been reported. This ND is made from carbon nanomaterials and is non-toxic and biocompatible [161]. Lung cancer cells have a surface protein called EGFR. Cisplatin is a chemotherapeutic agent that is also toxic for healthy cells when used alone, due to its ability to bind cisplatin inside by heparin. Packaging cisplatin in EGFR-targeted heparin nanoparticles aims to boost the drug’s tumor-killing power while cutting collateral damage; a smarter, gentler way to treat EGFR-positive lung cancer [162]. For the diagnosis of human lung adenocarcinoma A549 cells, a multifunctional nanostructure based on ferritin could be applied by fluorescence and MR imaging [163]. Porcine gelatin and human serum albumin are protein-based NPs are biocompatible; they have high cellular uptake and do not create inflammation in epithelial cells of the bronchial. Due to these characteristics, they are good for drugs and gene delivery [164]. A layer-by-layer assembly of polyelectrolytes on liposomes was created for the administration of PTX; this is because oral drug delivery to the pulmonary system has been impeded due to the reduced bioavailability of drugs. To overcome this problem, a layer-by-layer assembly of polyelectrolytes over liposomes was designed by Jain et al. in 2012 [156] for the administration of PTX. This PTX, along with stearyl amine, created the center of the NPs, which was additionally overlaid with successive layers of anionic poly (acrylic acid) (PAA) and then cationic poly (allylamine hydrochloride) (PAH). For the lung cancer treatment, lung adenocarcinoma cells (A549) were used to authenticate the effectiveness of the proposed system [165]. Lung cancer NPs and clinical trials against different models, along with their mechanisms, are shown in Table 2.

## 9. Nanoparticles in Prostate Cancer

Prostate cancer is one of the second most common cancers in men, especially in advanced age. There is a need for the development of a nanodevice for the early detection of prostate cancer and malignancy to overcome the lower specificity of chemotherapeutic agents. BIND-014, Docetaxel-loaded nanoprototype is an understudied option for prostate cancer [171]. Natural products like green tea, especially (−)-epigallocatechin 3-gallate (EGCG), showed a very good efficacy against induced carcinogenesis models [173,174,175]. In one of the preclinical trials, the EGCG was loaded in the NPs (polylactic acid-polyethylene glycol (PLA-PEG) and efficacy was measured [176]. In addition to this, A very novel EGCG was developed and encapsulated in NPs, which was further decorated with organic molecules of low molecular weight having targeted ligands ([poly(lactide-co-caprolactone-co-glycolide)-PEG, PLGA-PEG]) which bind with prostate-specific membrane antigens. These functionalized NPs showed a very selective efficacy against prostate membrane-specific antigen (PSMA) of prostate cells without affecting healthy cells in relative to NPs without functionalization [177,178]. In another study, EGCG as a chemopreventive agent was loaded on polysaccharide NPs for its delivery to the targeted cell [179]. Further investigation revealed that these NPs induced apoptosis mechanisms in prostate cells and, as a result, reduced their viability, which is a key process in the mitigation of cancer cells. Furthermore, lower concentration of encapsulated EGCG has an inhibitory effect on the proliferation of cancer cells compared to free EGCG, as exhibited by an in vitro assay.

In another experiment, it was reported that there was a huge reduction in prostate cell proliferation when it was treated with NPs (liposomes) containing curcumin (an anticancer agent), and these liposomes were already coated with prostate-specific membrane antigen antibodies [180]. In an in vitro study performed by Sanna et al., Dtx-loaded INPs were formulated along with two novel block copolymers, PLA-PCL (poly(lactide-co-caprolactone)) and PLGA-PCL (lactide-co-caprolactone-co-glycolide) in which it was exhibited that PLGA-PCL, along with Dtx-NPs, are more efficient against PCa cells compared to free Dtx after in vitro assay outcomes [181]. The size of the prostate gland and tumor can be reduced with the employment of the DT-A gene (diphtheria toxin suicide gene) having prostate-specific promoter to cells, while the direct injection of a gene without polymeric NPs does not affect the prostate gland and tumor either [182]. The prostate cells have a huge expression of HER2, which could be a good target to mitigate the PCa cells. Keeping the same concept in mind, Goldstein and his coworkers performed an experiment in which they combined the monoclonal antibody along with a chemotherapeutic agent (paclitaxel) and suggested a treatment option for prostate cancer [183].

For effective treatment of PCa, major attention was given to customized GNPs, magnetic NPs, and carbon nanotubes, which proficiently produce heat upon electromagnetic (light and magnetic fields) stimulation after direct injection or special buildup into tumors [184]. In preclinical cancer models, BIND-014, a nanomedicine, exhibited 20 times more Dtx delivered to the tumor as determined in one of the preclinical cancer model studies. This was a huge improvement in the tolerability and anticancer activity of Dtx in the tumor [185].

Epithelial cells from the prostate produce an antigen in serum; the name of the antigen is prostate-specific antigen (PSA). This is exclusively related to prostate cells’ physiology and pathophysiology and can be used as a biological marker of PCa. Bio-barcode developed by researchers can detect an undetectable level of PSA; this barcode is based on nanotechnology [186]. For the determination of circulating cancer cells in blood, Kattesh’s group attached GNPs to a prostate cancer cell line and detected them in a photoacoustic flowmeter [187]. In patients with recurrent PCa, thermotherapy was investigated through superparamagnetic iron oxide NPs (SPION) and evaluated an imaging-based calculation of the 3D-temperature distribution, a specific and noninvasive technique of magnetic fluid hyperthermia, and this was the first clinical application of interstitial hyperthermia by using magnetic NPs in patients with local recurrence of PCa [188]. Prostate cancer NPs and clinical trials against different models, along with their mechanisms, are shown in Table 3.

In clinical oncology, nanoparticle-based formulations such as liposomal doxorubicin, albumin-bound paclitaxel, liposomal irinotecan, liposomal vincristine, and CPX-351 have demonstrated the value of nanomedicine in improving pharmacokinetics, reducing systemic toxicity, and, in selected settings, improving therapeutic outcomes [79]. More recently, emerging platforms including lipid nanoparticle-based nucleic acid therapeutics and personalized mRNA cancer vaccines have shown encouraging early clinical results and are expanding the scope of nanoparticle applications beyond conventional chemotherapy [193]. Nevertheless, the clinical translation of nanoparticle therapeutics remains challenging because therapeutic performance often varies across patients and tumor types due to heterogeneous vascular permeability, stromal barriers, and immune clearance. Additional limitations such as large-scale manufacturing, batch-to-batch reproducibility, formulation stability, and regulatory complexity continue to slow the transition of promising nanoparticle systems from preclinical studies to routine clinical use [79,193,194,195].

The therapeutic performance of nanoparticles in breast, prostate, and lung cancers is strongly influenced by their size, shape, and surface properties. In breast cancer, smaller ligand-functionalized liposomes and polymeric nanoparticles (typically 50–150 nm) demonstrate improved penetration into dense tumor tissues and enhanced uptake by HER2- or folate receptor-positive cells [196]. In prostate cancer, surface-modified gold nanoparticles and polymeric nanocarriers conjugated with PSMA-targeting ligands improve selective accumulation and imaging sensitivity, while anisotropic shapes may enhance receptor-mediated internalization. In lung cancer, inhalable nanoparticles with optimized aerodynamic size and hydrophilic surface coatings show superior deposition in deep lung tissues, prolonged retention, and improved local drug bioavailability. Across these cancer types, positively tuned surface charge and tumor-specific ligands further enhance cellular internalization, therapeutic selectivity, and clinical response, highlighting the importance of tailoring nanoparticle physicochemical properties to the biology of each tumor type [196,197].

Although nanoparticle systems are often developed to reduce the systemic toxicity of conventional anticancer agents, their own safety profile requires equally critical consideration. Biocompatibility is strongly influenced by nanoparticle composition, size, surface charge, degradation products, and long-term biodistribution. Cationic and metallic nanoparticles may induce membrane damage, oxidative stress, mitochondrial dysfunction, inflammatory cytokine release, and complement activation, whereas poorly biodegradable systems may accumulate in the liver, spleen, lungs, or kidneys after repeated administration. In addition, protein corona formation may unpredictably alter cellular interactions and immunogenicity. Therefore, a balanced safety assessment must consider not only acute cytotoxicity but also chronic retention, organ-specific toxicity, biodegradation kinetics, and immunological compatibility before successful clinical translation can be achieved [16,198].

Despite the strong preclinical promise of nanoparticle-based therapeutics, successful clinical translation remains limited by several non-biological barriers. Regulatory approval is often challenging because multifunctional nanoparticle systems combine features of drugs, biologics, imaging agents, and devices, creating uncertainty in classification and safety evaluation pathways. In addition, large-scale manufacturing requires strict control of particle size distribution, drug loading efficiency, sterility, shelf stability, and batch-to-batch reproducibility, which may be difficult to maintain for complex multifunctional formulations. Economic factors further complicate translation, as advanced nanoparticle synthesis, purification, surface modification, and quality control processes substantially increase production costs compared with conventional formulations. Therefore, scalability, regulatory simplicity, and cost-effectiveness are now recognized to be equally critical as therapeutic efficacy in determining whether a nanoparticle platform can realistically progress from laboratory innovation to routine oncology practice [16,29].

A critical comparison across nanoparticle platforms indicates that clinical success is determined not solely by anticancer efficacy, but by the balance between therapeutic performance, safety, stability, and translational practicality. Lipid-based nanoparticles currently show the strongest clinical impact due to their reproducible manufacturing, favorable safety profiles, and regulatory familiarity, whereas polymeric nanoparticles offer superior control over release kinetics and multifunctional engineering. In contrast, inorganic and highly complex multifunctional nanoparticles often demonstrate excellent imaging or theranostic capabilities in preclinical studies but remain limited by long-term retention, uncertain biodegradability, and scale-up complexity. These comparative insights emphasize that the future value of nanoparticle systems lies in rational simplification and mechanism-aware optimization rather than increasing structural sophistication alone.

Recent advances in nanocarrier engineering further emphasize that successful clinical translation depends on overcoming biological transport barriers, particularly limited deep tumor penetration and heterogeneous microenvironmental conditions. Emerging strategies such as charge-reversal surfaces, size-transformable nanocarriers, and tumor microenvironment-responsive systems have shown strong potential to improve intratumoral distribution and therapeutic precision. In addition, recent translational frameworks highlight that manufacturability, regulatory simplicity, and biological reproducibility remain equally important determinants of clinical success, underscoring the need for rational nanoparticle design that balances complexity with translational feasibility [199,200].

Combination therapy using nanoparticles offers major therapeutic advantages, but its clinical application remains challenging because co-loaded agents often differ in solubility, stability, pharmacokinetics, and optimal dose ratios [56]. To address these issues, current strategies focus on designing multifunctional or compartmentalized nanoparticles that allow synchronized or sequential release, surface-targeted delivery, and stimulus-responsive drug unloading within the tumor microenvironment. In addition, toxicity management is being improved through tumor-selective ligands, reduction in off-target exposure, use of biocompatible carriers, and optimization of drug-loading ratios to preserve synergy while minimizing dose-limiting adverse effects. These approaches may improve the safety and efficacy of nanoparticle-based combination therapies, particularly in tumors requiring multimodal treatment [49,56,201].

## 10. Conclusions and Future Perspective

Cancer remains one of the leading causes of premature mortality worldwide, underscoring the urgent need for more effective and targeted therapeutic strategies. This review highlights that, despite significant advances, conventional cancer therapies continue to be limited by systemic toxicity, poor tumor selectivity, and reduced efficacy in advanced disease stages. In contrast, nanoparticle-based systems have emerged as a promising platform to overcome these challenges through improved tumor targeting, controlled drug release, and multifunctional capabilities.

A key finding of this review is that nanoparticle performance is highly dependent on both material composition and biological context. Lipid-based and polymeric nanoparticles currently demonstrate the greatest clinical relevance due to their biocompatibility, tunable release profiles, and successful translation into approved formulations, whereas inorganic nanoparticles provide distinct advantages in imaging, photothermal therapy, and theranostic applications. However, their clinical performance remains inconsistent across tumor types.

Importantly, this review identifies several critical limitations that continue to hinder the clinical translation of nanoparticle-based therapies. These include tumor heterogeneity, variability in EPR- and ATR-mediated delivery, protein corona formation, immune clearance, and challenges related to large-scale manufacturing, regulatory approval, and cost. These factors collectively contribute to variability in therapeutic outcomes and limit reproducibility across patient populations.

Future research should move beyond generalized nanoparticle design and focus on tumor-specific and patient-tailored strategies. Priorities include integrating tumor microenvironment characteristics into nanoparticle engineering, improving delivery efficiency through biologically informed transport mechanisms, developing standardized frameworks for toxicity and long-term safety evaluation, and advancing scalable and reproducible manufacturing processes. Addressing these challenges will be essential to bridge the gap between experimental promises and clinical implementation of nanomedicine in oncology.

## Figures and Tables

**Table 1:** Breast NPs and their clinical trials against different models.

Nanoparticle Type/Size	Target/Model	Mechanism	Clinical Stage	References
Doxil^®^ (PEGylated liposomal doxorubicin)/~100 nm	Metastatic/recurrent breast cancer	PEGylated liposome: passive EPR accumulation and doxorubicin release in the tumor	Approved by the FDA	[138]
Abraxane^®^ (albumin-bound paclitaxel, nab-PTX)/∼130 nm	Metastatic/early age breast cancer	Albumin nanoparticle exploits gp60 & SPARC-mediated transcytosis; solvent-free paclitaxel	Approved by the FDA	[139,140,141]
MM-302 (HER2-targeted liposomal doxorubicin)/≈85 nm	HER2-positive metastatic BC	Anti-HER2 scFv-grafted liposome—receptor-mediated endocytosis	Phase 2 completed	[142]
CRLX101 (cyclodextrin-polymer ⟶ camptothecin)/25–35 nm	Triple-negative BC xenograft	Cyclodextrin-PEG polyplex releases CPT in acidic tumor micro-environment	Under phase 2	[143,144,145]
Genexol-PM^®^ (polymeric micellar paclitaxel)/20–50 nm	Metastatic breast cancer	mPEG-PLA micelle enhances paclitaxel solubility; EPR uptake	Phase 3	[146]
Au-PSMA aptamer nano-gel + siRNA/60–80 nm	Orthotopic TNBC	Gold nano shell gel delivers siPLK1; photothermal & gene silencing	Pre-clinical	[147]

**Table 2:** Lung NPs and their clinical trials against different models.

Nanoparticle Type/Size	Target/Model	Mechanism	Clinical Stage	References
Lipusu^®^ (liposomal paclitaxel)/80–100 nm	NSCLC (China)	Stealth liposomal paclitaxel, solvent-free; passive targeting	Approved by CFPA	[166]
BIND-014 (PSMA-target docetaxel NP)/70–90 nm	PSMA-positive NSCLC	Active PSMA targeting + docetaxel payload	Phase II halted	[167]
NC6300 (epirubicin micelle)/80 nm	Advanced NSCLC cohort	PEG-poly(aspartate) micelle carrying epirubicin	Phase 1	[168]
SiGNa-TPGS/SN38 NP/110 nm	EGFR-mut H1975 model	Silica–γ-Fe_2_O_3_ NP with TPGS matrix; SN38 delivery and ROS	Pre-clinical	[169,170]
SPION-Gefitinib magnetic NP/50 nm	Gefitinib-resistant H1975	Superparamagnetic iron-oxide core + gefitinib; magnetic targeting & EGFR inhibition	Pre-clinical	[171]
CRLX101 (CPT polymeric NP)/25–35 nm	Recurrent NSCLC	Hypoxia-activated CPT prodrug NP	Phase 2	[172]

**Table 3:** Prostate cancer NPs and their clinical trials against different models.

Nanoparticle Type/Size	Target/Model	Mechanism	Clinical Stage	References
BIND-014—PSMA-targeted PLA-PEG nanoparticle carrying docetaxel/70–90 nm	PSMA-positive metastatic castration-resistant prostate cancer (mCRPC)	Active binding to PSMA on prostate-tumor endothelium—endocytosis and intratumor docetaxel release	Phase 11	[167]
PSMA-aptamer/PLGA–doxorubicin NP (≈150 nm)	LNCaP xenograft (PSMA^+^)	Aptamer-guided uptake; PLGA core gives sustained DOX release	Pre-clinical	[189]
Cabazitaxel-PLGA NP (≈120 nm)	DU-145 xenograft (CRPC)	Biodegradable depot—sustained cabazitaxel release, ↑ tumor AUC	Pre-clinical	[190]
CRLX101—cyclodextrin-PEG camptothecin NP (25–35 nm)	Docetaxel-resistant PC-3 xenograft	EPR accumulation; pH/hypoxia-triggered CPT release—Topo-I inhibition	Pre-clinical (PC); Phase II other tumors	[133]
Ionisable-lipid LNP–siRNA (AR-NTD), 70–100 nm	22Rv1 xenograft (androgen-independent)	LNP delivers siRNA against AR N-terminal domain—blocks AR signaling	Pre-clinical	[191]
CV9104 RNActive^®^ multi-antigen mRNA–LNP vaccine (≈80 nm)	Biochemical-recurrent & metastatic CRPC (Phase IIb, NCT02111577)	LNP delivers mRNA encoding PSA, PSMA, PSCA, STEAP1, PAP, MUC1—poly-antigen T-cell response	Phase IIb (completed)	[192]

## Data Availability

No data was used for the research described in the article.
